# Analytical Detection of Sulfonamides and Organophosphorus Insecticide Residues in Fish in Taiwan

**DOI:** 10.3390/molecules25071501

**Published:** 2020-03-25

**Authors:** Chung-Pei Chang, Po-Hsun Hou, Wei-Cheng Yang, Ching-Fen Wu, Chia-Chia Chang, Ming-Yang Tsai, Hsiao-Pei Tsai, Chien-Teng Lin, Yi-Jing Xue, Jiann-Hsiung Wang, Geng-Ruei Chang

**Affiliations:** 1 Department of Anesthesiology, Show Chwan Memorial Hospital, 1 Section, 542 Chung-Shan Road, Changhua 50008, Taiwan; fentanylpei@yahoo.com.tw; 2 Department of Psychiatry, Taichung Veterans General Hospital, 4 Section, 1650 Taiwan Boulevard, Taichung 40705, Taiwan; peterhopo2@yahoo.com.tw; 3 Faculty of Medicine, National Yang-Ming University, 2 Section, 155 Linong Street, Beitou District, Taipei 11221, Taiwan; 4 Department of Veterinary Medicine, School of Veterinary Medicine, National Taiwan University, 4 Section. 1 Roosevelt Road, Taipei 10617, Taiwan; yangweicheng@ntu.edu.tw; 5 Department of Veterinary Medicine, National Chiayi University, 580 Xinmin Road, Chiayi 60054, Taiwan; cfwu@mail.ncyu.edu.tw (C.-F.W.); vet540423@gmail.com (C.-T.L.); asdzxc0523@gmail.com (Y.-J.X.); jhwang@mail.ncyu.edu.tw (J.-H.W.); 6 Animal Drugs Inspection Branch, Animal Health Research Institute, Council of Agriculture, 21 Muchang, Ciding Village, Zhunan Township, Miaoli County 35054, Taiwan; bccchia@mail.nvri.gov.tw; 7 Animal Industry Division, Livestock Research Institute, Council of Agriculture, Executive Yuan, 112 Muchang, Xinhua Dist, Tainan 71246, Taiwan; mytsai@mail.tlri.gov.tw; 8 Graduate Institute of Bioresources, National Pingtung University of Science and Technology, 1 Shuefu Road, Neipu, Pingtung 91201, Taiwan; 9 Veterinary Teaching Hospital, National Chiayi University, 580 Xinmin Road, Chiayi 60054, Taiwan; tsaibelle@mail.ncyu.edu.tw; 10 Ph.D. Program of Agriculture Science, National Chiayi University, 300 Syuefu Road, Chiayi 60004, Taiwan

**Keywords:** sulfonamide, organophosphorus insecticide, residue, fish, risk assessment

## Abstract

Exposure to residues of antibiotics (e.g., sulfonamides) and insecticides (e.g., organophosphorus insecticides) in aquacultured food can adversely affect humans and animals and thus affect public health globally. Here, using a validated method, we examined the levels of residues of 12 sulfonamides as well as 18 organophosphorus insecticides in aquacultured fish in Taiwan. A total of 52 fish samples (i.e., 20 tilapia, 16 milk fish, and 16 perch samples) were obtained from Taiwanese aquafarms from June 2018 to October 2019. We detected 0.02 and 0.03 mg/kg of sulfamethazine (a sulfonamide) in one tilapia and one milk fish, respectively, and 0.02, 0.05, and 0.03 mg/kg of chlorpyrifos (an organophosphorus insecticide) in one tilapia, one milk fish, and one perch, respectively; thus, among the samples, 3.85% and 5.77% contained sulfonamides and organophosphorus insecticide residues, respectively. Furthermore, we assessed human health risk based on the estimated daily intakes (EDIs) of these residues: EDIs of sulfonamide and organophosphorus insecticide residues were <1.0% of the acceptable daily intake recommended by the Joint Food and Agriculture Organization of the United Nations/World Health Organization Expert Committee on Food Additives. The risk of exposure to sulfonamide and organophosphorus insecticide residue by consuming aquacultured fish in Taiwan was thus negligible, signifying no immediate health risk related to the consumption of fish. Our findings can constitute a reference in efforts geared toward ensuring food safety and monitoring veterinary drug and insecticide residue levels in aquacultured organisms. Residue levels in fish must be continually monitored to further determine possible effects of these residues on human health.

## 1. Introduction

The aquaculture industry is of great economic importance and has been growing faster than other animal-farming industry worldwide [1]. Taiwan’s environment and geographic location are highly appropriate for aquaculture advancement, with the country’s aquaculture history spanning more than three centuries [2]. In Taiwan, more than 35 major and candidate aquatic species are commercially aquacultured [3]. Over 2010–2018, Taiwan’s revenue derived from land aquaculture was up to US$1 billion on average [3]; in addition, during the 2010s, Taiwan’s estimated annual land aquaculture production was 300,000 t [4]. Moreover, in Taiwan, major developments in aquaculture occurred over the 1960s to the 1990s [2], mainly because of the strong support of the government since the 1960s [5]. In particular, the government strongly supported the farming of fish, such as tilapia, milkfish, perch, eels, and groupers. From the beginning of tilapia farming in 1978 until 2018, Taiwan’s average annual tilapia production increased to 60,000 t [3].

Taiwan has a subtropical climate and a limited amount of usable land; thus, aquaculture farms in Taiwan are located close to agricultural and residential areas, predisposing the cultured organisms to fungal, bacterial, parasitic, and viral infections. This thus necessitates applying prophylactic and therapeutic veterinary drugs, such as antibiotics [6]. Nevertheless, excessively applying these drugs could result in the cultured organisms retaining drug residues at harvest, potentially exposing consumers to substances toxic for human consumption [2]. We previously detected chloramphenicol in hard clam [6] and shrimp [2] and quinolones in shrimp [2] aquafarms in Taiwan. Moreover, these aquafarms use several insecticides to control ectoparasites and endoparasite growth [7] and several pesticides to limit the growth of aquatic weeds in bodies that include canals, fishponds, and lakes [6]. These activities lead to the introduction of these chemicals into water and soil and consequently contaminate aquaculture regions. Aquaculture techniques that are usually executed in Taiwan’s inner regions include polyculturing with waterfowls and mixed breeding with different aquatic products; this may also introduce chemical pollution to the soil as well as water environments and thus negatively affect aquacultured organisms [8]. In aquaculture, excessively applying chemicals may engender public health concerns as well as ecological impacts; for example, bacterial resistance could be induced and chemical residues may be added to the environment, thus increasing allergy and cancer risks. Therefore, research should be executed on whether aquacultured fish contain veterinary drug and insecticide residues—possibly bioaccumulated in their edible parts.

The recent continual decrease in the availability of wild-caught fish has led to a rise in aquaculture fish production as well as a rise in their consumption. However, in Taiwan, major food safety incidents over the past decade have been related to aquatic product consumption [9] and economic losses have been incurred because the import of some Taiwanese aquacultured food products has been banned by several countries worldwide [10]. Consequently, Taiwan’s government is paying increasing attention toward aquacultured product safety and quality assessment and management. Sulfonamides, synthetic derivatives of sulfonic acid, are used broadly because they are low-cost, are effective against some bacterial infections, and improve animal performance. In the Asia Pacific region, sulfonamides are commonly used against bacterial infections and other diseases in aquaculture [1]. In China, for instance, sulfur drugs were detected in all fish samples from typical marine aquaculture regions [11]. However, sulfonamides have several side effects; for example, they have been demonstrated to engender reduced filet palatability of aquacultured products, increased kidney damage and infertility risk, and physiological and immunological response disruptions [1,12]. Massive and repeated illegal use of organophosphorus insecticides to control parasitic diseases in aquacultured organisms has also been noted in Taiwan [4]; these insecticides mainly inhibit acetylcholinesterase activity and disrupt nerve function in humans [13].

The Taiwan Food and Drug Administration (TFDA) has yet to establish the maximum residue limits (MRLs) for sulfa drugs (except for sulfadimethoxine and sulfamonomethoxine) or organophosphorus insecticides in fish. However, in 2019, the TFDA defined the MRLs of sulfa drugs in livestock, chicken, milk, and eggs and completely banned the use of sulfa drugs in fish aquaculture. Therefore, in this study, we determined the concentrations and accumulation levels of sulfonamides and organophosphorus insecticides in major aquacultured fish in Taiwan. In addition, we examined these contaminants’ estimated daily intake (EDI) through seafood consumption in Taiwanese adults. Our findings provide information that can be potentially useful during the development of effective measures for safe aquaculture and aquacultured product consumption. Moreover, our results highlight the potential phthalate burden imposed on consumers due to excessive plastic material use in Taiwan.

## 2. Results

### 2.1. Detected Levels as Well as Rates of Residues of Sulfonamides in Analyzed Fish Samples

In total, 52 fish samples were analyzed. Table 1 presents the detected levels of banned sulfonamide residues in different fish samples. In all fish samples, the predominant sulfonamide residue was sulfamethazine (Figure S1): 5% in tilapia (1/20; concentration: 0.03 mg/kg) and 6.25% in milk fish (1/16; concentration: 0.02 mg/kg). The data for the remaining 19 tilapia, 15 milk fish, and 16 perch samples, which did not demonstrate detectable levels of sulfonamides, are not shown. Moreover, on average, 0.002, 0.001, and 0 mg/kg sulfonamides were detected in all tilapia, milk fish, and perch samples, respectively. Finally, only 2 (3.85%) of all 52 samples contained sulfonamides (i.e., sulfamethazine).

### 2.2. Detection Levels and Rates of Organophosphorus Insecticide Residues in Fish Samples

Table 2 presents the detected levels of the residues of prohibited organophosphorus insecticides in different fish samples. In all fish samples, the predominant organophosphorus insecticide residue was of chlorpyrifos (Figure S2): 5% in tilapia (1/20; concentration: 0.02 mg/kg), 6.25% in milk fish (1/16; concentration: 0.05 mg/kg), and 6.25% in perch (1/16; concentration: 0.03 mg/kg). The data of the remaining 19 tilapia, 15 milk fish, and 16 perch samples, which did not demonstrate detectable levels of organophosphorus insecticides, are not shown. That is, in general, the detected levels of chlorpyrifos were between 0.02 and 0.05 mg/kg. Moreover, on average, 0.001, 0.003, and 0.002 mg/kg chlorpyrifos were detected in all tilapia, milk fish, and perch samples, respectively. Finally, only 3 (5.77%) of all 52 samples contained sulfonamides (i.e., sulfamethazine).

### 2.3. EDI Levels of Sulfonamide Residues Through Fish in Taiwanese Adults

Average EDI levels extrapolated from average sulfamethazine levels in Taiwanese women and men were 0.001 and 0.002 μg/kg body weight/day, respectively (Table 3). The acceptable daily intake (ADI) level of sulfamethazine residues through food is 0.05 mg/kg, as stipulated by the Joint Food and Agriculture Organization of the United Nations/World Health Organization Expert Committee on Food Additives (JECFA) [1,14]. The obtained EDI levels were determined to be substantially lower than the JECFA-recommended ADI levels of sulfamethazine (Table 3). Moreover, the EDI levels expressed as the percentages of the ADIs were 0.004% and 0.002% in men and women respectively. Taken together, fish consumption led to a low risk assessment for sulfamethazine, with EDIs that are <0.1% of the ADIs in both men as well as women.

### 2.4. EDI Levels of Residues of Organophosphorus Insecticides Ingested Through Fish in Taiwanese Adults

The average EDI levels extrapolated from the average chlorpyrifos levels in Taiwanese women and men were 0.002 and 0.003 μg/kg body weight/day, respectively (Table 4). The EDI levels were noted to be substantially lower than the JECFA-recommended chlorpyrifos ADI level of 0.01 mg/kg (Table 4) [4,15]. Moreover, the EDI levels expressed as the percentages of the ADIs were 0.03% and 0.02% in men and women, respectively. Taken together, fish consumption led to a low risk assessment for chlorpyrifos, with EDIs that are <0.1% of the ADIs in men as well as women.

## 3. Discussion

We analyzed 52 fish samples from aquaculture regions in Taiwan for the residues of 12 sulfonamides (i.e., sulfamerazine, sulfaethoxypyridazine, sulfathiazole, sulfadiazine, sulfamethoxypyridazine, sulfapyridine, sulfadoxine, sulfamethazine, sulfadimethoxine, sulfamethoxazole, sulfamonomethoxine, and sulfameter,) and 18 organophosphorus insecticides (i.e., chlorfenvinphos, chlorpyrifos, diazinon, fenamiphos, fenitrothion, fenthion, formothion, iprobenfos, malathion, methacrifos, methamidophos, methidathion, phoxim, profenophos, prothiofos, pyrazophos, triazophos, and trichlorfon). The TFDA allows the presence of only sulfadimethoxine and sulfamonomethoxine in aquacultured products at total residual levels of <0.1 mg/kg and prohibits the use of any other sulfa drugs and organophosphorus insecticides in fish aquaculture. These compounds’ residues existing in fish were thus identified; moreover, the identified levels of the residues were noted to adequately demonstrate the level of legal compliance concerning the application of these products.

The limit of quantification (LOQ) of all sulfonamides in fish samples was 10 ng/g—identical to the LOQ recommended by the TFDA for sulfonamide contamination in edible livestock, chicken, milk, and aquacultured foods and half of that for sulfonamide contamination in animal viscera [16]. However, the TFDA has not recommended a strict LOQ for organophosphorus insecticide contamination in aquacultured foods. Nevertheless, the TFDA-recommended LOQ is 10 ng/g for organophosphorus insecticide contamination in livestock (e.g., pork) and chicken muscles [17]. Previous methods developed for analyzing organophosphorus insecticide contamination in bivalves, crustaceans, fish, and cephalopods have involved the use of various apparatuses. For instance, gas chromatography flame ionization revealed the LOQs of chlopyriphos, ethion, ethoprophos, fensulfothion, isoxathion, and parathion to be 2–50 ng/g [18]; gas chromatography–mass spectrometry revealed the LOQs of propetamphos, diazinon, disulfoton, malathion, fenthion, and triazophos to be 7–15.2 ng/g [19]; and high-performance liquid chromatography (HPLC)–tandem mass spectrometry (LC-MS/MS) coupled with gel permeation chromatography revealed the LOQs of profenofos, chlorpyrifos, malathion, phosmet, triazophos, trichlorphon, and dimethoate to be 0.05–0.2 ng/g [20]. Compared with the aforementioned LOQs, our present analytical method detected a lower LOQ (i.e., 5 ng/g) for organophosphorus insecticide multiresidues, indicating the usefulness of our method in detecting trace organophosphorus insecticide residues.

Here, we adopted the analytical approach for sulfonamide residues suggested by the TFDA [16] and that for organophosphorus insecticide residues developed by the European Committee for Standardization, the QuEChERS (quick, easy, cheap, effective, rugged, and safe) method [4,21]—both of which are suitable for detecting trace chemical residues. For the validation of its analytic method, the TFDA [2,22] recommends an acceptable recovery rate of 70–120% [relative standard deviation (RSD) < 15%], 70–120% (RSD < 20%), and 50–125% (RSD < 35%) for chemical residues in food matrices detected in the 0.1–1, 0.001–0.01, and <0.001 mg/kg ranges, respectively. In this study, residues of sulfonamides and organophosphorus insecticides that were identified within the 0.01–0.1 mg/kg ranges were noted to exhibit a recovery rate of 90–120% (RSD < 15%) and 80–120% (RSD < 15%), respectively. Moreover, the quantities of spiked analytes used at the lower and higher levels were respectively 5 and 25 ng/g for the 12 sulfonamides (Table S3) and 10 and 50 ng/g for the 18 organophosphorus insecticides (Table S4). Thus, all analyzed sulfonamide and organophosphorus insecticide concentrations were within the acceptable range [22]. Accordingly, our method of extraction proposed herein was robust for the analysis of the investigated sulfonamides and organophosphorus insecticides in the fish samples.

LC-MS/MS demonstrated a positive result for sulfonamide in only 3.85% of the 52 fish samples, and in all positive samples, only sulfamethazine, a TFDA-banned sulfonamide, was observed. Sulfonamide residues were identified in 77.59% of a total of 116 samples of fish in 2016 in China [23], 4.27% of 101 samples of fish over 1994–1998 in Slovenia [24], 17.39% of 138 samples of fish in 2012 in Iran [25], and 1.75% of 171 samples of fish over May September 2008 in South Korea (specifically sulfadiazine and sulfamethoxazole residues) [26]. The TFDA conducted surveys for sulfonamide residues in aquacultured products over 2013–2018 in Taiwan and detected increased illegal use of sulfonamides as antibiotic agents in marine products in order to increase production [27,28,29,30,31,32]: the detection rate of banned sulfonamide residues in fish samples was 0.50% in 2013 (1/199) [27], 0% in 2014 (0/194) [28], 4.29% in 2015 (3/70) [29], 1.49% in 2016 (1/67) [30], 2.94% in 2017 (2/68) [31], and 2.67% in 2018 (2/75) [32]. However, our current findings are inconsistent with the aforementioned findings from the TFDA surveys. These differences may possibly be due to varied sample sizes. In addition, our study samples were derived from areas of fish production in Taiwan; by contrast, those derived by the TFDA could have been imported fish. Moreover, we included a larger fish sample size and analyzed for more sulfonamides. Therefore, a wider spectrum of banned sulfonamide residues was analyzed in fish in Taiwan.

The predominant sulfonamide residue was sulfamethazine, with its maximum concentration being 0.03 mg/kg (in one tilapia sample). This is consistent with the results observed by Sampaio et al. [1] and Nunes et al. [14]: sulfamethazine is a frequently applied sulfonamide in fish aquaculture. Moreover, sulfamethazine has previously been found to be present at high concentrations (>100 μg/kg) frequently [23]. The aforementioned studies have positively identified sulfathiazole [27], sulfamethoxazole [29,30,32], and sulfadiazine [31] in fish samples, and a TFDA survey positively identified sulfaquinoxaline and sulfathiazole in soft-shell turtle samples [28]. These findings confirm the increased use of sulfonamides as feed additives in aquaculture [11]. In addition, the water sulfamethazine level of 100 ng/L could affect greenhouse gas release, atmospheric ozone depletion, and eutrophication control through denitrification inhibition and N_2_O release stimulation [33]. Moreover, high sulfonamide usage appears to be strongly correlated with bacterial resistance to sulfonamides [34], achieved through the development of antibiotic resistance genes, which can pose a potential human health risk [23]. Our results, in combination the findings derived from other surveys described here, demonstrate the exposure of Taiwan’s population to trace sulfonamide levels through fish consumption.

Of all 18 analyzed organophosphorus insecticides, only chlorpyrifos was detected in the fish samples. In all fish samples, 5.77% was the detection rate for all studied organophosphorus insecticide residues, with the highest rate being for chlorpyrifos (6.25%) in milk fish and perch samples, followed by tilapia samples (5.0%). During their aquaculture activities, some farmers in Taiwan apply organophosphorus insecticides for ectoparasite treatment [4]. Sun et al. [35] reported an organophosphorus insecticide detection rate of 11.37% in 607 fish samples from traditional markets, regional supermarkets, fish markets, and fish farms in Taiwan over 2001–2003; however, over 2002–2004, the authors reported an organophosphorus insecticide detection rate of 16.83% (only chlorpyrifos) in 814 fish samples from Taiwan markets [36]. Our results concerning organophosphorus insecticide detection rates are much lower than those of Sun et al. [35,36]. These discrepancies are likely due to differences in the sample sizes and collection sources as well as the differences in the organophosphorus insecticide categories analyzed. These could also have occurred because of the Taiwanese government’s implementation of the national action plan for reducing veterinary drug use in 2006 [37].

The predominant organophosphorus insecticide in our fish samples was chlorpyrifos—similar to the observations of a similar survey in Egypt [13]. Even though chlorpyrifos rapidly degrades in the environment, the Egyptian study reported that extensive chlorpyrifos use polluted aquatic habitats and caused increased general toxicity to all vertebrates compared with other classes of insecticides. Chlorpyrifos remains a widely used organophosphorus insecticide for controlling pests in agriculture as well as in sanitation industries around the globe [38]. Because of neurotoxicity concerns, Taiwan’s government has banned the use of organophosphorus insecticides (except for trichlorfon) in aquaculture and specified strict MRLs for animal husbandry [4]. In the current study, the insecticides detected may have been illegally applied for the direct treatment of ectoparasite infections occurring in fish or for the treatment of infectious diseases caused by the presence of other aquacultured organisms during mixed breeding in aquaculture ponds, as we revealed previously [4,6,7]. Moreover, we detected chlorpyrifos levels of 0.002 mg/kg, which are much lower than the level revealed by Sun et al. (0.463 mg/kg) [36]. The aquacultured fish also exhibited more variation in chlorpyrifos residues. The chlorpyrifos contamination sources could be fish feed and aquaculture environments, including water and sediments. A study reported that chlorpyrifos rapidly degrades in the marine ecosystem [39], but data on chlorpyrifos contamination in fish feed are limited. Thus, we recommend additional studies examining phthalate content in fish feeds. Information on organophosphorus insecticide use for ectoparasite infection treatment in other special fish species, however, remains limited. The detected levels of organophosphorus insecticide varied among all three fish species; this variation is attributable to differences in the sample sizes used. We thus recommend the execution of further research employing large samples of cultured fish.

Several guidelines provide parameters that can aid in assessing human and other organisms’ risks of various conditions after exposure to chemical residues through food; these parameters include ADI, target hazard quotient (THQ), and tolerable daily intake (TDI) [2,4,40]. ADI is recommended by JECFA, but apparent discrepancies in consumption rates and eating habits are not considered in ADI [7]. Through the use of TDI, one could evaluate the risks that are related to consuming specific food products tainted with plasticizers and could identify whether exposure to plasticizers such as phthalates at various degrees is harmful to human health [41]. THQ indicates the assessment of health risk of noncarcinogenic harmful effects [40]. The JECFA [42] and US Environmental Protection Agency [43] have proposed EDI, which can be used to estimate chronic dietary intake at a relatively high accuracy level. In the current study, EDIs were not exceeded by the corresponding ADIs. Because few sulfonamide residues were detected, the EDIs revealed that fish consumption led to a substantially lower dietary intake of sulfamethazine, relative to that specified by the corresponding ADIs, in Taiwan’s population. In addition, when evaluated against the ADIs, the EDI corresponded to <1% of the ADIs, signifying that the corresponding risk is negligible [2,7,44]. Farmers engaged in aquaculture in Taiwan may also apply organophosphorus insecticides for ectoparasite treatment [4,45]. In the present study, the consumption of aquacultured fish led to exposure to very low levels of the organophosphorus insecticide chlorpyrifos: in the Taiwanese population, its EDI was much lower than its JECFA-recommended ADI (<0.1% of the ADI for organophosphorus insecticide residues). Therefore, these results were similar to those for sulfamethazine for fish consumption. The risk assessment is negligible because the EDI is <1% of the corresponding ADI [2,7,44]. Taken together, sulfonamide and organophosphorus insecticide residue levels in aquacultured fish in Taiwan may not affect human health adversely.

Our results suggest that to ensure commercial food safety, regulatory authorities as well as producers in Taiwan must endeavor to continually monitor aquacultured products and potential contamination sources. Moreover, considering that antibiotics may exert adverse effects on health and aquatic environments, additional studies on the effects exerted by such pollutants are imperative.

## 4. Materials and Methods

### 4.1. Samples, Chemicals, and Reagents

In total, 52 fish samples (20 tilapia, 16 milkfish, and 16 perch samples) were collected from Taiwanese aquafarms in major production areas located in Yunlin, Chiayi, Tainan, Kaohsiung, and Pingtung over June 2018 to October 2019 (Figure S3). As surveyed in 2018 by Fisheries Agency of Taiwan, these fish were bred on a large scale in Taiwan [3]. From the obtained fish samples (each weighing ~1200 g and comprising 2 fish), fish muscles were extracted, followed homogenization and storage at −20 °C until analysis. In the analyses executed in this study, three replicates were extracted from each composite sample to determine each tested compound’s average concentration.

Sigma-Aldrich (St. Louis, MO, USA) was the source of the analytical compound standards of the 12 sulfonamides used in this study, namely sulfamethazine, sulfathiazole, sulfamethoxazole, sulfamethoxypyridazine, sulfadoxine, sulfamerazine, sulfaethoxypyridazine, sulfameter, sulfamonomethoxine, sulfadimethoxine, sulfapyridine, and sulfadiazine (all >95.0% purity). Moreover, those of organophosphorus insecticide, namely chlorfenvinphos, chlorpyrifos, diazinon, fenitrothion, fenthion, malathion, methacrifos, methamidophos, methidathion, phoxim, profenophos, pyrazophos, and trichlorfon (all >97.0% purity) were from Dr. Ehrenstorfer GmbH (Augsburg, Germany). In addition, fenamiphos (99.5% purity) were obtained from Bayer CropScience AG (Monheim, Germany), formothion (96.0% purity) from Sandoz India (Mumbai, India), iprobenfos (99.6% purity) from Sigma-Aldrich, and prothiofos (95.5% purity) and triazophos (99.5% purity) from ChemService (West Chester, PA, USA). Merck (Darmstadt, Germany) was the source of chromatography-grade acetone, anhydrous sodium sulfate (ASS), methanol (MeOH), formic acid (FA), ethyl acetate, n-pentane, n-hexane, and acetonitrile (ACN). We also purchased, from Agilent Technologies (Wilmington, DE, USA), 15-mL QuEChERS cleanup tubes (Agilent SampliQ QuEChERS EN fatty dispersive-SPE kit, p/n 5982-5156) and a QuEChERS extraction salt packet (Agilent SampliQ QuEChERS EN Extraction kit, p/n 5982-5650; mixture constituents: 1 g sodium citrate, 1 g NaCl, 0.5 g citric acid disodium salt, and 4 g anhydrous magnesium sulfate).

### 4.2. Instruments and Apparatus

A nitrogen evaporator (N-Evap-111; Organomation Associates, Berlin, MA, USA), centrifuge (Allegra X-22R; Beckman Coulter, Fullerton, CA, USA), nitrogen generator (Model 05B, System Instruments Co., Tokyo, Japan), and vortex mixer (type 37,600 mixer, Barnstead Thermolyne, Dubuque, IA, USA) were employed in this study for sample preparation. For LC-amenable sulfonamides, LC-MS/MS consisted of a mass spectrometer (ABI 4000 QTRAP, Applied Biosystems, Foster City, CA, USA) in electrospray ionization (ESI) mode and an UltiMate 3000 HPLC system (Thermo Fisher Scientific, Waltham, MA, USA). HPLC separation was executed using an Acquity UPLC^®^ HSS T3 C18 column (2.1 mm internal diameter × 1.8 μm film thickness ×100 mm; Waters, Milford, MA, USA) with chromatographic conditions at 40 °C. To determine the levels of residues of LC-amenable organophosphorus insecticides, the liquid chromatography–tandem mass spectrometry (LC-MS/MS) apparatus comprised an UltiMate 3000 HPLC system and a mass spectrometer (TSQ quantiva triple quadrupole; Thermo Fisher Scientific, Austin, TX USA). In addition, LC-amenable organophosphorus insecticides were separated using a CORTECS UPLC C18 column (2.1 mm internal diameter × 1.6 μm film thickness ×100 mm; Waters, Milford, MA, USA). To analyze gas chromatography (GC)-amenable organophosphorus insecticides, GC-tandem mass spectrometry (GC-MS/MS) was executed on a GC system (Thermo Scientific Trace 1310; Thermo Fisher Scientific, Austin, TX USA) as well as a mass spectrometer (TSQ 8000 triple quadrupole, Thermo Fisher Scientific) that was coupled with Rxi^®^-5Sil MS fused silica capillary column (0.25 mm internal diameter × 0.25 μm film thickness × 30 m; Restek, Bellefonte, PA, USA).

### 4.3. Standard Solution Preparation

To prepare stock solutions of individual sulfonamides and organophosphorus insecticide standards in this study, a portion of each analyte (100 mg) was accurately weighed and dissolved in volumetric flasks containing 100 mL of acetone, ACN, or MeOH, according to analyte solubility. For the preparation of working standard mixtures, all stock solution types were combined and subsequently diluted to 1 mg/L. For storage, all solutions were maintained at −20 °C; however, before each use, they were brought to room temperature. The prepared working standard solutions were applied to derive a series of calibration standards through serial dilution in the 1–500 ng/mL range.

### 4.4. Sample Preparation and Extraction

To detect sulfonamide residues, we used TFDA’s directions for multiresidue analysis of residues of veterinary drugs in foods, which required the cleaning and homogenization of fish samples first [46]. In brief, 5 g of the homogenate and 25 mL of ACN in 5% MeOH were mixed on a vortex mixer for 3 min. Then, 10 g of ASS was added to the homogenate and mixed for 10 min; subsequently, centrifugation was executed at 3500 g at 4 °C for 10 min, after which only the supernatant layer was retained. The remaining tissue pellets were re-extracted using 25 mL of ACN in 5% MeOH, followed by centrifugation. The first extract was combined with the previously separated ACN layer. The resulting mixture was subjected to liquid–liquid extraction in a separating funnel. The filtrate was then added to 30 mL of ACN-saturated n-hexane and mixed on a vortex mixer for 10 min. The ACN-extracted layer was dried at 40 °C in a nitrogen evaporator. The evaporation residue was dissolved in 1 mL of 50% MeOH and filtered through a 0.2-µm polyvinylidene fluoride filter (Whatman, Maidstone, UK). Next, the derived filtrate was transferred to an autosampler vial before injection into a chromatograph.

For analyzing residues of insecticides in fish samples, we used the European Committee for Standardization-developed QuEChERS extraction procedure [6,7,47]. In brief, 10 g of homogenized fish sample and 10 mL of ACN were mixed vigorously in a 50-mL centrifuge tube for 1 min; then, QuEChERS extraction salt was added, and the mixture was mixed on a vortex for 1 min and then subjected to a 5-min centrifugation process executed at 3000 g. Thereafter, crude ACN extract (~6 mL) was transferred into QuEChERS cleanup tubes. The ACN layer was mixed vigorously for 2 min and centrifuged at 3000 g for 5 min. Next, 1 mL of the extract was filtered through a 0.2-μm filter membrane and transferred into an autosampler vial for LC-MS/MS. Another 1 mL of extracted solution was near-completely dried in the nitrogen evaporator at 40 °C. Finally, the residue was redissolved with 1 mL of a 1:1 (v/v) n-hexane and acetone mixture, filtered through a 0.2-μm filter membrane, and introduced into an autosampler vial for GC-MS/MS.

### 4.5. LC-MS/MS Parameters

The injection volume used for detecting the sulfonamide and organophosphorus insecticide residues was 10 µL. The mobile phase was binary, comprising eluents A (0.1% FA) and B (0.1% FA in MeOH), and the gradient of the mobile phase was developed as follows: 5% eluent B from 0 to 2 min (flow rate, 0.3 mL/min); followed by a step increase of eluent B to 20% from 2 to 3 min, 25% from 3 to 6 min, 27% from 6 to 8.6 min, and 37% from 8.6 to 14.5 min; then linear increase to 100% eluent B from 14.5 to 14.7 min; and finally decrease to 4% eluent B at 18.7 min, which was maintained from 18.7 to 20 min. MS was determined in positive ESI modes with monitoring of the two most abundant MS/MS (precursor/product) ion transitions by using an MRM program for each analyte. The MS parameter settings are outlined as follows: collision gas argon pressure, 0.12 mL/min; desolvation flow, 1000 L/h; source temperature, 150 °C; desolvation temperature, 500 °C; dwell time for every MRM transition, 5 ms; cone gas flow, 50 L/h; capillary voltage, 3 kV. Table S1 lists the precursor and corresponding product ions with optimum collision energy obtained through the MRM detection for 12 sulfonamides and LC-amenable 6 organophosphorus insecticides.

### 4.6. GC-MS/MS Parameters

GC-MS/MS analysis was executed in positive and negative electron-impact ionization interface modes. The carrier gas, namely helium, was applied at a constant flow rate of 1 mL/min. The temperatures of injector were 280 °C. In addition, the oven temperature was set at 60 °C—it was initially maintained isothermal for 1 min, next raised to 170 °C at 40 °C/min, and finally maintained at 310 °C for 8 min. The set source and transfer-line temperatures were 300 and 250 °C, respectively. In the splitless mode, the injection volume was determined to be 10.0 μL. In the collision chamber (second quadrupole), these ions were collision-activated with argon at 4.4 mTorr. Table S2 lists the precursor and corresponding product ions with optimum collision energy obtained through the MRM detection for GC-amenable 12 organophosphorus insecticides.

### 4.7. Quality Assurance and Validation

To validate our described method, recovery, repeatability, linearity, and LOQ were estimated [2,4,6]. For repeatability and recovery estimation, we spiked blank samples (in triplicate) with a standard mixture of the analytes at two concentrations 5 (low) and 25 (high) ng/g for analysis of sulfonamides and 10 (low) and 50 (high) ng/g for analysis of LC- and GC-amenable organophosphorus insecticides. The recovery was then calculated through the comparison of the noted concentrations of samples spiked before extraction with blanks spiked at the same concentration after extraction.

The derived reproducibility is presented herein as RSD (%); moreover, the LOQs were derived as the analyte concentration that generated a peak signal 3–10 times higher than the background noise in the chromatogram. For evaluation of linearity, matrix-matched calibration was executed by using blank sample extracts and then adding the corresponding amount of the working target compound solution at concentrations of 1–500 ng/mL. Linearity of the calibration curves was detected by fitting least-squares regression analysis in a linear mode (R^2^ ≥ 0.990) in the studied concentration range. All sample concentrations lower than their corresponding LOQs were regarded as undetectable [4,7,47].

### 4.8. Sulfonamide and Organophosphorus Insecticide EDIs Compared with ADIs

To assess the degree of exposure to veterinary drug residues through fish in Taiwanese people, the corresponding EDIs of the sulfonamide and organophosphorus insecticide residues were determined. The JECFA-recommended ADIs for these residues were used for comparison. EDI calculation was executed as follows [2,6,7]:

EDI (ng/kg/day) = [(daily fish consumption in g/day) × (mean veterinary drug concentration in ng/g)]/(human body weight in kg)

Moreover, data regarding Taiwan residents’ daily seafood consumption were extracted from the results of the National Nutrition and Health Survey by Ministry of Health and Welfare, Taiwan: 96.9 g for men and 74.2 g for women [48]. The mean Taiwanese body weight was considered to be 60 kg [48]. The maximal EDIs were determined from maximum residue concentrations.

## 5. Conclusions

We report an efficient and sensitive LC and GC-MS/MS-based method for detecting sulfonamide and organophosphorus insecticide residues in fish. The most abundant and the only sulfonamide and organophosphorus insecticide residues detected in all fish samples were sulfamethazine and chlorpyrifos, respectively. Nevertheless, all the detected chemicals were present in trace amounts, much below the TFDA-recommended MRLs, in all 52 fish samples. This indicates that the presence of sulfamethazine and chlorpyrifos use during large-scale breeding of fish in Taiwan does not lead to severe contamination. Furthermore, EDIs for these chemicals in Taiwanese adults were considerably lower than the JECFA-defined ADIs—confirming that no immediate health risk is posed by consuming the aquacultured fish. Therefore, the low sulfamethazine and chlorpyrifos intake through the consumption of contaminated fish in Taiwan seems to present a negligible threat to the health of Taiwanese people. Taiwanese regulatory authorities and aquafarmers may use our findings as a reference for improving aquaculture-related food safety regulation.

We, however, are unaware of contaminants other than sulfonamides and organophosphorus insecticides used in fish production that may be consumed during daily meat intake and may exceed their ADIs to a hazardous extent within the general population. Regardless of our findings, global concern regarding veterinary antibiotic and insecticide contamination and adverse effects on the environment and human health is increasing. Thus, a background information system on veterinary antibiotic and insecticide consumption through fish must be established and improved so as to provide an appropriate monitoring and management framework. Moreover, aquatic samples should be continually surveyed for detecting residues of chemicals and ensuring food safety.

## Figures and Tables

**Table 1:** Detected levels of residues of prohibited sulfonamides in fish samples collected over June 2018 to October 2019.

Fish	Targets Detected	Surveyed Samples	No. of Residue	Detected Residues ^1^ (mg/kg)	Average ^2^ (mg/kg)	Residual Ration ^3^ (%)
Tilapia	sulfamethazine	20	1	0.03 ± 0.0003	0.002 ± 0.0015	5
Milk fish	sulfamethazine	16	1	0.02 ± 0.0005	0.001 ± 0.0010	6.25
Perch	undetected	16	0	0	0	0
Total	sulfamethazine	52	2	0.02–0.03	0.001 ± 0.0006	3.85

^1^ Values are given as means ± SEM. ^2^ Estimated for all samples (detected and not detected to have sulfonamide residues). ^3^ Samples with residual concentrations lower than the limit of quantification were considered to have undetectable concentrations.

**Table 2:** Detected levels of residues of organophosphorus insecticides in fish samples obtained over June 2018 to October 2019.

Fish	Targets Detected	Surveyed Samples	No. of Residue	Detected Residues ^1^ (mg/kg)	Average ^2^ (mg/kg)	Residual Ration ^3^ (%)
Tilapia	Chlorpyrifos	20	1	0.02 ± 0.0003	0.001 ± 0.0010	5.0
Milk fish	Chlorpyrifos	16	1	0.05 ± 0.0006	0.003 ± 0.0030	6.25
perch	Chlorpyrifos	16	1	0.03 ± 0.0003	0.002 ± 0.0018	6.25
Total	Chlorpyrifos	52	3	0.02–0.05	0.002 ± 0.0011	5.77

^1^ Values are given as means ± SEM. ^2^ Estimated for all samples (detected and not detected to have organophosphorus insecticide residues). ^3^ Samples with residual concentrations lower than the limit of quantification were considered to have undetectable concentrations.

**Table 3:** Estimated daily intake (EDI) levels of sulfonamide residues in Taiwanese adults.

Sulfonamides	EDI (μg/kg Body Weight/Day) Weight/Day Weight/Day)		EDI% of ADI		ADI (JECFA) (mg/kg Body Weight/Day)
	Male	Female	Male	Female	
sulfamethazine	0.002	0.001	0.004	0.002	0.05

**Table 4:** EDI levels of organophosphorus insecticide residues in Taiwanese adults.

Insecticides	EDI (μg/kg Body Weight/Day) Weight/Day Weight/Day)		EDI% of ADI		ADI (JECFA) (mg/kg Body Weight/Day)
	Male	Female	Male	Female	
Chlorpyrifos	0.003	0.002	0.03	0.02	0.01

## Supplementary Materials

The following are available online, Table S1: MS/MS fragmentation conditions for 12 sulfonamides and LC-amenable 6 organophosphorus insecticides. Table S2: MS/MS fragmentation conditions for GC-amenable 12 organophosphorus insecticides. Table S3: Recovery, repeatability, and limit of quantification of veterinary drugs spiked into tilapia samples. Table S4: Recovery, repeatability, and limit of quantification of organophosphorus insecticides spiked into tilapia samples. Figure S1: LC-MS/MS chromatogram of the detected 12 sulfonamides residues at the quantification ion for sulfamethazine in the positive samples. Figure S2: GC–MS/MS chromatograms of the detected 18 organophosphorus insecticide residues at the quantification ion for chlorpyrifos in the positive samples. Figure S3: Location of 52 sampling areas in Taiwan.
