# Supplementary material for: Analytical Detection of Sulfonamides and Organophosphorus Insecticide Residues in Fish in Taiwan
Source: Molecules. 2020 Mar 25;25(7):1501. doi: 10.3390/molecules25071501 (PMC7181026; doi:10.3390/molecules25071501)
Supplement: Supplementary file 1 [file molecules-25-01501-s001.pdf]

Table S1. MS/MS fragmentation conditions for 12 sulfonamides and LC-amenable 6 organophosphorus insecticides

Analyte	Retention Time	Quantification ion (Collision energy) m/z (eV)	Identification ion (Collision energy) m/z (eV)
Sulfadiazine	5.41±0.02	251→156 (16)	251→92 (19)
Sulfadimethoxine	14.74±0.02	311→156 (8)	311→92 (18)
Sulfadoxine	10.63±0.02	311→156 (28)	311→92 (7)
Sulfaethoxypyridazine	12.68±0.02	295→156 (11)	295→92 (8)
Sulfamerazine	6.62±0.02	265→156 (10)	265→92 (6)
Sulfameter	7.64±0.02	281→156 (10)	281→92 (4)
Sulfamethazine	8.07±0.02	279→156 (17)	279→186 (5)
Sulfamethoxazole	9.38±0.02	254→156 (34)	254→92 (17)
Sulfamethoxypyridazine	8.44±0.02	281→156 (16)	281→92 (6)
Sulfamonomethoxine	9.62±0.02	281→156 (16)	281→92 (6)
Sulfapyridine	6.16±0.02	250→156 (10)	250→92 (6)
Sulfathiazole	5.84±0.02	256→156 (11)	256→92 (6)
Fenamiphos	10.87±0.02	304→202 (37)	304→217 (24)
Fenthion	9.63±0.02	279→169 (20)	279→247 (14)
Methamidophos	2.83±0.02	142→94 (14)	142→125 (13)
Phoxim	11.30±0.02	299→129 (10)	299→153 (10)
Profenophos	11.65±0.02	373→128 (55)	373→303 (19)
Trichlorfon	4.93±0.02	257→79(28)	257→109 (18)

Table S2. MS/MS fragmentation conditions for GC-amenable 12 organophosphorus insecticides

Analyte	Retention Time	Quantification ion (Collision energy) m/z (eV)	Identification ion (Collision energy) m/z (eV)
Chlorfenvinphos	11.10±0.02	267→159 (20)	323→267 (15)
Chlorpyrifos	10.33±0.02	314→258 (15)	314→286 (5)
Diazinon	8.64±0.02	304→179 (15)	304→162 (5)
Fenitrothion	10.05±0.02	277→109 (20)	277→260 (5)
Formothion	9.24±0.02	224→125 (20)	224→155 (10)
Iprobenfos	9.13±0.02	204→91 (10)	204→122 (15)
Malathion	10.16±0.02	173→127 (5)	173→99 (15)
Methacrifos	8.14±0.02	125→79 (5)	125→62 (5)
Methidathion	11.51±0.02	145→85 (5)	145→58 (15)
Prothiofos	12.02±0.02	267→239 (10)	267→221 (20)
Pyrazophos	15.61±0.02	221→193 (10)	232→204 (15)
Triazophos	13.17±0.02	257→162 (5)	257→119 (30)

Table S3. Recovery, repeatability, and limit of quantification of sulfonamides spiked into tilapia samples

Analyte	Tilapia			
	spiked level (ng/g)	Recovery (%)	RSD (%)	LOQ (ng/g)
Sulfadiazine	5	90.52	9.37	10
	25	98.21	12.65	
Sulfadimethoxine	5	93.32	3.06	10
	25	95.67	3.82	
Sulfadoxine	5	105.76	10.69	10
	25	112.51	11.53	
Sulfaethoxypyridazine	5	108.52	1.53	10
	25	105.67	3.48	
Sulfamerazine	5	92.37	8.95	10
	25	95.75	6.72	
Sulfameter	5	106.57	2.86	10
	25	108.51	6.58	
Sulfamethazine	5	95.68	2.43	10
	25	96.18	1.48	
Sulfamethoxazole	5	92.39	2.11	10
	25	95.65	4.28	
Sulfamethoxypyridazine	5	102.85	7.63	10
	25	105.38	2.13	
Sulfamonomethoxine	5	99.36	5.48	10
	25	101.53	7.62	
Sulfapyridine	5	100.75	7.45	10
	25	102.31	4.53	
Sulfathiazole	5	100.82	5.73	10
	25	105.36	9.25	

Table S4. Recovery, repeatability, and limit of quantification of organophosphorus insecticides spiked into tilapia samples

Analyte	LC/GC-amenable	Tilapia			
		spiked level (ng/g)	Recovery (%)	RSD (%)	LOQ (ng/g)
Chlorfenvinphos	GC	10	102.82	6.51	5
		50	117.23	7.83	
Chlorpyrifos	GC	10	92.46	7.11	5
		50	98.57	5.63	
Diazinon	GC	10	102.39	5.62	5
		50	108.35	6.14	
Fenamiphos	LC	10	105.36	2.32	5
		50	108.74	3.19	
Fenitrothion	GC	10	98.31	6.13	5
		50	95.62	5.32	
Fenthion	LC	10	108.65	6.58	5
		50	117.82	4.89	
Formothion	GC	10	105.72	8.62	5
		50	108.61	2.57	
Iprobenfos	GC	10	104.35	4.58	5
		50	108.54	7.52	
Malathion	GC	10	103.12	10.71	5
		50	116.28	11.52	
Methacrifos	GC	10	82.15	14.56	5
		50	92.73	16.12	
Methamidophos	LC	10	81.05	1.28	5
		50	83.24	2.59	
Methidathion	GC	10	104.82	5.32	5
		50	112.67	7.51	
Phoxim	LC	10	117.86	1.25	5
		50	118.63	2.52	
Profenophos	LC	10	109.18	1.15	5
		50	112.32	2.86	
Prothiofos	GC	10	107.83	5.26	5
		50	115.76	4.32	

Table S4. continued

Analyte	LC/GC-amenable	Tilapia			
		spikd level (ng/g)	Recovery (%)	RSD (%)	LOQ (ng/g)
Pyrazophos	GC	10	102.63	9.57	5
		50	117.81	5.67	
Triazophos	GC	10	115.27	7.52	5
		50	118.25	6.23	
Trichlorfon	LC	10	98.92	3.42	5
		50	102.86	5.65	

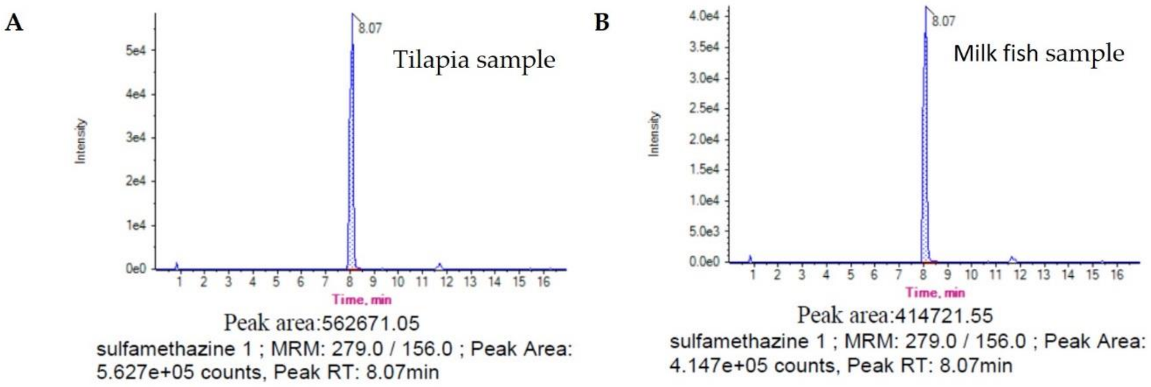

Figure S1: LC-MS/MS chromatogram of the detected 12 sulfonamides residues at the quantification ion for sulfamethazine in the positive samples

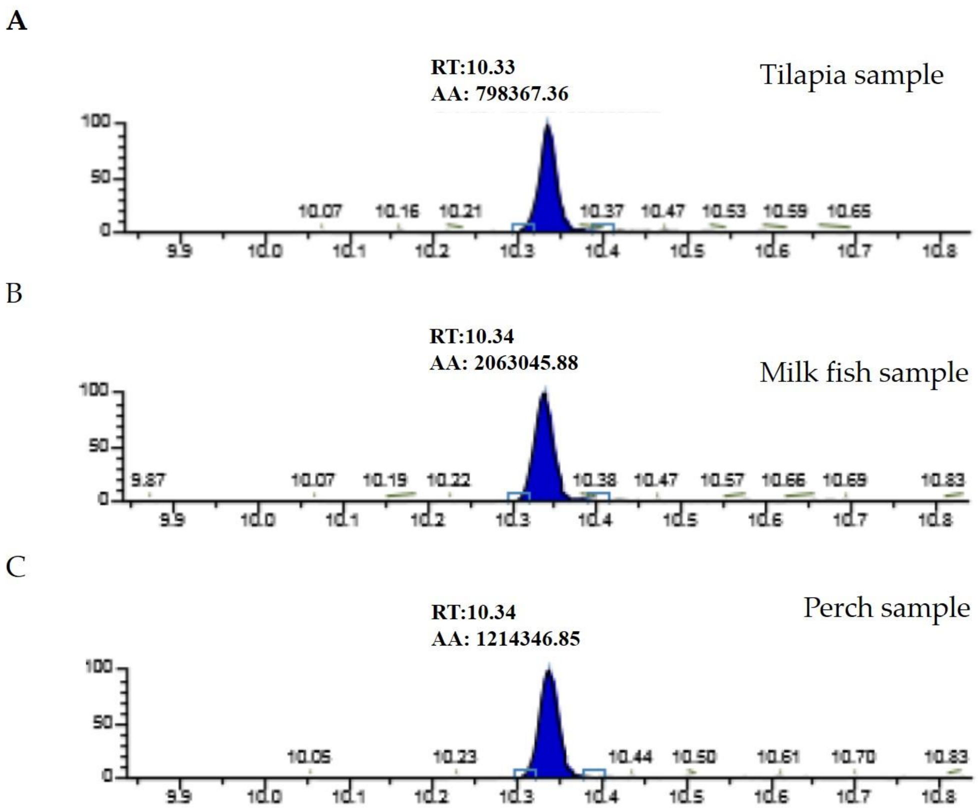

Figure S2: GC–MS/MS chromatograms of the detected 18 organophosphorus insecticide residues at the quantification ion for chlorpyrifos in the positive samples.

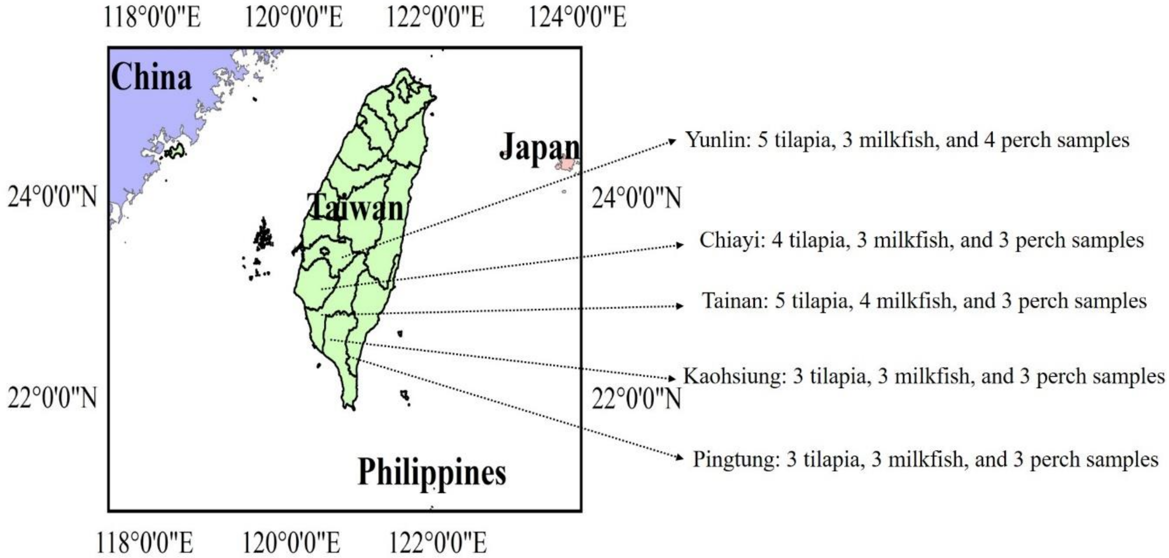

Figure S3: Location of 52 sampling areas in Taiwan.
